# The New Education and Its Teachers

**Published:** 1917-05-26

**Authors:** 


### SOME PROBLEMS OF TO-DAY.*

### V. The New Education and its Teachers.

We have become accustomed to large numbers. Millions of men, hundreds of thousands of casualties, a billion in money—items once almost unthinkable are now matters of ordinary calculation and conversation. When, for instance, we read that Mr. Fisher will require some forty to fifty thousand new teachers for the development of the new education, and that the mere raising of the school age to a universal fourteen would at once call for the addition of 4,000 or 5,000, we are interested rather than dismayed. When about £4,000,000 in additional expenditure is asked for, we agree that the teachers' salaries certainly ought to be raised, and that no doubt any money remaining is needed for very good purposes. Scarcely a grudging voice has been raised to declare, as of old, that "they teach 'em a deal too much," and that education is already unfitting the elementary school child for manual labour.

The vast additional expense of the new education is therefore hardly to be called a problem. Popular clamour may demand that the Treasury rather than local rates shall furnish the money. Even the question of compulsion seems likely to be amicably settled. A long battle will rage over curricula for adolescents and the organisation in detail of nursery schools. But the question of man—and woman—power will be a serious one.

The latest suggestion made to the University woman, who finds herself in astonishingly high demand at present, is that she should go into trade. It is true that the offer, as it stands, is somewhat indefinite, but there is something attractive about this new field of work in which great fortunes are not seldom made by the other sex. The lecture given by Miss Burlton on May 4 to the students of University College, London, exemplified by her own success in one of the best-known drapery firms in London, was received and commented on with evident interest. Meantime some high authorities have been expressing the opinion that a woman of University standing is wasted on the primary school. The Jesuit's exceptionally long training taught him to consider the small child his most valuable pupil, since the foundations of character could best be laid before the age of seven. The educated woman may surely do well to consider, before she throws herself into trade, whether her abilities are of a kind which might help to further the extremely important national experiment of the new nursery school. We assume, of course, that something entirely different from anything hitherto established in this country is intended. Not a miniature copy of the ordinary school is promised, but a nursery in which the infant's budding powers of body and mind are to be nursed to full strength by the development of healthy physical and moral dispositions and habits. Not acquirement of knowledge, but the discipline of the growing mind is to be aimed at, tending to ready initiative, self-control, mutual helpfulness, interest in surroundings, cheerful good temper. Observation on the part of the teacher will give the child freedom of choice in occupation, on the Montessori system, and physical exercise and tactile training will figure largely in the curriculum. Folk-dance and game and song will certainly be drawn upon, and as the study of infancy and infant mentality proceeds we may look for much application of new ideas if the managers and teachers are intelligent persons in love with their work. Otherwise a dreary stereotyping of routine must ensue.

But such an experiment demands teachers not merely intelligent but gifted and trained. The unintelligent and untrained applications of theories of educational freedom may too easily degenerate into licence, while physical care and development, as now understood, demand skill and knowledge which the ordinary teacher, however fond of children and interested in them, has had no opportunity to acquire. Neither, probably, has the University woman. Where, then, shall we look for the considerable number of suitable women to be very soon needed?

Some good material, surely, should be found amongst girls who have taken up V.A.D. work, and have given it up, perhaps because of the discouraging conditions of the early times, perhaps for want of bodily strength. There are also women medical students who find the strain too great, but have acquired some knowledge and give up their training with reluctance. There are also some young girls and some in "second girlhood" who have worked as voluntary helpers in mother-schools and welfare centres, and who would gladly pass on to more organised and salaried work. Many of these are now engaged in clerical and other work in which their former experiences are wasted. It is true that the present urgent appeal for more V.A.D. candidates will call out some of them, but it is a pity if those who have real love for children and a gift for teaching are not now secured and trained, especially in the science of teaching and the control of numbers.

At the other end of the scale, longer school life and combination classes will offer a great opportunity to those who "have always said" that "children should not be allowed to grow up in ignorance of such-and-such things." We have an uncomfortable recollection of the stuffed vegetables in our old friend of the nursery bookshelf, "The Water Babies." The choice of subjects and the *privilege* of teaching them to the older boys and girls who have reached the age when children of the more educated class are just beginning to learn—these are tasks surely worthy of some of the best brains in the country. There is yet a third opening. Mr. Fisher has just told us that his first act on entering the Board of Education was to invite school managers in manufacturing towns to establish play-centres. The Board now will provide half the expenses of such centres, and they are fast springing up all over the country. In America several Universities already provide training for teachers who will specialise in this work. Again, this is work worth brain power. Why, by the way, do we think chiefly of the brains of the University *woman*?

Some thirty-five years ago a few men of good birth with University degrees went into the primary schools. Very few then persevered, the conditions, of course, were much inferior to what they are now. The time is ripe for a fresh effort of this kind. Nothing would so rejuvenate popular education as a similar and larger movement now, at once raising the status of the elementary school master and widening his outlook, besides improving his salary. After the war, or, indeed, before its end, many men with University degrees will seek work possible to their partly incapacitated physique. Their experience will make them heroes in the eyes of all boys. May we hope for some as recruits in the new army of the soldiers of Peace?

* Previous articles appeared March 31, April 7, May 5 and 19.

